# Older residents’ perceptions of family involvement in residential care

**DOI:** 10.1080/17482631.2019.1611298

**Published:** 2019-05-09

**Authors:** Sarah Sio Wa Lao, Lisa Pau Le Low, Kayla Ka Yin Wong

**Affiliations:** a Kiang Wu Hospital, Macau, Macao SAR; b School of Health Sciences, Caritas Institute of Higher Education, Tseung Kwan O, Hong Kong

**Keywords:** Family involvement, Macao Chinese, older people, residential care homes, qualitative

## Abstract

**Aim:** This study explored the Chinese older people's perceptions regarding family involvement and specific factors influencing family involvement in residential care homes. **Background:** Family involvement in residential care home is a multi-dimensional construct that connects the resident with their family, friends, neighbours and care professionals to facilitate their physical, psycho-emotional and social well-being. However, it remains unclear as to what Chinese residents perceive as the meaning of involving the family and components of it that are important to later live. **Material and Methods:** A descriptive qualitative study using semi-structured interviews was conducted in two residential care homes in Macao. Ten Chinese residents were purposively sampled. The interview data were taped-recorded and transcribed. Fieldnotes and visitors' records were collected. The data were content-analyzed. **Results:** Chinese residents' perceptions of family involvement were captured by four themes: components of family involvement, factors influencing family involvement, impacts of family involvement on residents' lives, and promoting family involvement strategies. **Conclusion:** Findings provided insights for geriatric care providers to acknowledge the contributions that family members can make to be more involved in the residents' live, and to strengthen relationships. Family involvement can also help to facilitate sense of blessing and feelings of achievements for the residents.

## Introduction

The involvement of family members in the lives of older people following entry into residential care homes has gained increasing attention over the decades. Bowers (1988) purported that family involvement of older residents should focus on maintaining family connectedness, promoting the older person’s recovery and participating in the care home environment. Given the limited data available to illustrate the circumstances of family involvement in residential care homes in the Chinese context, a classical statement from the Federal Nursing Home Reform Act (1987) is cited here to highlight the forms and roles of the family, and that the establishment of family councils in government long-term care institutions had served the purposes of improving the residents’ quality of life and care. This can enable family members to contribute to decision-making by facilitating effective communication between themselves and the homes (Gaugler, 2005). Therefore, family involvement has impeccable importance in connecting the residents with their family members, friends, neighbours and healthcare professionals to promote their physical, psycho-emotional and social well-being (Bern-Klug & Forbes-Thompson, 2008; Buckley & McCarthy, 2009; Ryan & Scullion, 2000; Whitaker, 2009). It also extends bonding with adult children to maintain parent–children relationship, perform filial piety and adopt positive attitudes to continue with caring responsibilities (Kellet, 2007; Mass et al., 2004). However, it appears that residents are likely to become disconnected with their social circles after they have been admitted into the homes (Teeri, Leino-Kilpi, & Välimäki, 2006).

**Figure 1.**
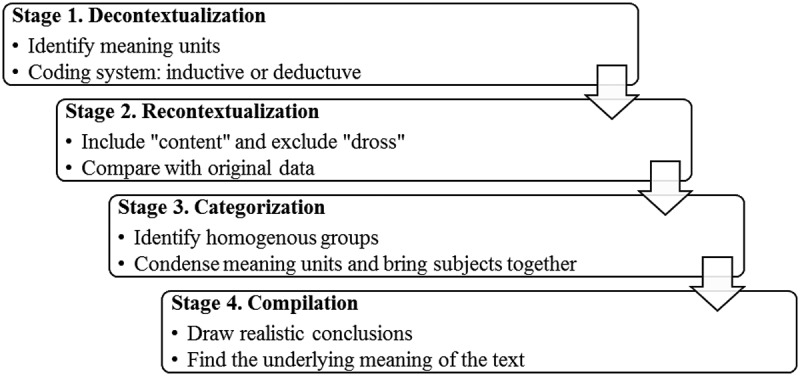
Process of data analysis.

Gaugler (2005) defined family involvement as a multidimensional construct that entails visiting, socio-emotional care, advocacy, and the provision of personal care. The family member was identified as a caring partner, and a resident’s representative who could work together with health-care providers to meet health and safety needs, and to attain the greatest degree of personal fulfilment and human dignity (Commonwealth of Australia, 2013; World Health Organization, 2002). This suggests that family involvement involves collaborating with the residents, family and care providers in order to construct a win-win situation for all the stakeholder groups concerned. Consequently, the inclusion of family members in the older person’s later life should be highly encouraged.

The literature depicted that family involvement in residential care homes can be categorized into direct care and indirect care. Direct care included providing simple bodily care, accompanying, delivering family or social messages, sharing meals and times together, and assisting in daily living activities and instrumental activities of daily living (Bern-Klug & Forbes-Thompson, 2008; Buckley & McCarthy, 2009; Whitaker, 2009). Conversely, indirect care included supervising and monitoring by the family during visits and showing their engagement in ensuring the residents’ health condition and ongoing care. Moreover, some family members attended care conferences to understand the lives of older residents and to speak up for them to facilitate better care in the homes (Bauer, 2006; Bern-Klug & Forbes-Thompson, 2008). For example, a quasi-experimental study from Taiwan implemented a videoconference programme using communicative software (either MSN or Skype) to promote resident–family interactions (at least 5 minutes per week, once a week and lasting for 3 months) in the homes had demonstrated significantly higher scores in emotional and appraisal social support, and lower scores in loneliness and depressive status (Tsai, Tsai, Wang, Chang, & Chu, 2010). Other factors that encouraged the frequency and duration of family involvement were found in older residents who had shorter length of stay at the home, had higher-dependency care and abled to remember the visits. Family members who were busy, encountered transportation issues, had poorer health status, inadequate social support, and poorer resident–family–staff relationship were found to be barriers affecting them from being involved at the homes (Fukahori et al., 2006; Gaugler, Anderson, & Leach, 2003; Natan, 2008; Port, 2004).

In the Chinese society, Confucian is regarded as the fundamental values for morals and social interactions which deeply affect the caring interactions between older parents and their family members. Filial piety influences how the adult children take on the responsibility to look after their older parents. Tsai and Tsai (2012) found that honouring filial piety responsibility, maintaining family relationships, and hoping for physical and emotional recovery for the residents were family members’ perceived meaning of the visits at the homes. A Confucian proverb “obey your children when you are old” can influence an older person’s attitude and expectations towards their children, and views towards family and social support as one essential component of successful ageing (Hsu, 2007). Other researchers have revealed the Chinese values of harmony, balance and collectivism (Lee, 1999), and a consideration for the psycho-emotional support of care as a component of the quality of family care (Chao & Roth, 2005). These studies point to the variations in the underlying meaning of family involvement in residential care homes from a Western and Chinese perspective. Indeed, a fundamental Confucianism philosophy, filial piety, is a virtue of respect for one’s parents and elders in the Chinese culture. A study found that filial piety influences how the family takes care of their elders by considering their functional utilities and socio-psychological outcomes in Taiwan, Hong Kong and China (Yeh, Yi, Tsao, & Wan, 2013). Unlike Western families, Chinese adult children are struggling to manage the cultural stigma with the financial, caregiving and emotional concerns of caring for their elders in the family. Health status, age and family resources are major factors influencing the decision to move into institutions. In the Chinese context, the continuing roles of family members in long-term care continue to be important after the placement of their elders in residential care homes (Zhan, Xiaotian, & Baozhen, 2008).

Among the Chinese population, there is currently no specific data to illustrate the circumstances of family involvement in residential care homes, and the overseas literature may not be correspondingly suitable to explain the situation for the Chinese residents. In Macao, the profile of older people admitted into the homes is on an increase, with reasons including those who were unable to live independently and had poorer health conditions that required nursing care (Social Welfare Department of Macao SAR Government, 2008). Given that the number of senior population (aged 65 years and over) has reached over 68,900, representing 10.5% of the total population in 2017 (Statistics & Census Service of Macao SAR Government, 2017), the number of homes has also increased from 15 in 1999 to 18 in 2018. There was a corresponding increase in the number of residents from 967 in 1999 to 1,023 in 2016 (Social Welfare Bureau Social Welfare Bureau of Macao SAR Government, 2018; Statistics & Census Service of Macao SAR Government, 2017). Consequently, it is estimated that the senior population in need of long-term care will rise to 16.0% by 2026. In response to the ageing population, the Government has announced the Ten-year Action Plan for the Provision of Services for the Elderly for 2016–2025. It also established an inter-departmental study group to implement 421 short-term, medium-term and long-term measures to address four aspects of a related policy framework. These aspects were healthcare and social services, protection of rights, social engagement, and living environment (Information Bureau of Macao SAR Government, 2017; Statistics & Census Service of Macao SAR Government, 2017).

The continuous care provided by the family would change family care arrangements after older people reside in residential care homes. The creation of a caring partnership between family and staff can work towards a home-like and professional care environment to provide a high standard of resident care. A descriptive qualitative study was conducted to understand the meaning of Chinese older people’s perceptions regarding family involvement in residential care homes of Macao. Specific factors influencing the degree of family involvement will be explored to help develop resident-oriented care with specific cultural elements. This study is the first study disclosing the situation of family involvement for Chinese family in Macao.

## Material and methods

### Study design

A descriptive qualitative methodology was selected in order to enter into a person’s world and to discover the wisdom and possibility of achieving the best understanding of the meaning and nature of family involvement from the older resident’s perceptions (Creswell, 2007). Streubert, Speziale and Carpenter (2011) indicated that perception is a way of observing and processing things that are present to the self. Each individual’s interpretation is different and is based on what that person perceives to be the reality.

### Settings and participants

Two governmental-sponsored homes in Macao were conveniently selected with a capacity ranging from around 40 to 170 (Social Welfare Bureau of Macao SAR Government, 2018). Both homes were non-profit and governmental-sponsored institutions. These homes provided regular meals, personal care, basic medical and nursing care, and social support for residents with mild to serious physical or mental limitations in activities of daily living but were suitable for communal living (Social Welfare Bureau of Macao SAR Government, 2018). An on-site coordinator with healthcare background referred potential participants to the researcher. Ten Chinese older residents (five from each home) were recruited by purposive sampling (Palys, 2008). The inclusion criteria were residents who had resided for at least six months, aged 65 or above, able to speak Cantonese, available to give written consent, and rated by the Cantonese Mini Mental State Examination (CMMSE) (Palys, 2008). Based on the original validation study of the CMMSE (Chiu, Lee, Chung, & Kwong, 1994), the tool has been modified, translated and validated from the original MMSE with satisfactory reliability (a = 0.86, RC = 0.78 and IRR = 0.99) and validity (CC = 0.94 and high discriminant percentage of 97.9%). According to Chui et al. (1998), three cut-off points to identify cognitive impairment were adopted in this study. These were a score of ≥ 18 points for the illiterate elders, ≥ 20 points with 1–2 years of education, and ≥ 22 points with more than 2 years of education. Older residents diagnosed with cognitive or mental impairment were excluded.

Of 10 participants recruited, 2 were female and 8 male with an age range from 68 to 94 years (average age of 78.2). The average mean score of performance in ADLs measured by Barthel Index was 58 (range 15–100). The average length of stay at the home was 27.9 months. Six of them completed primary school education, two completed senior high school, and two received no education. For the “primary” family caregivers, six were female, eight of them were children of the residents and the other two were wives. Their average age was 45.3 years (majority ranging from 22–53 years, with one being 72 years). The health status was rated as good and seven were still working. For the mode of transportation to the homes, five family members came on foot and five of them by private vehicles. The frequency of visits varied from daily to monthly, with three families visited daily, one visited four times per week, and six made weekly or less than a weekly visit. There were no major difference between gender and frequency of visits. However, the family members’ age, health status, occupation and mode of transportation to the home influenced the frequency of visits.

### Ethical considerations

Ethical approval was obtained from the university concerned. Permission was also sought from the administrative personnel-in-charge of each home. Following the provision of a clear explanation to each participant as well as their rights to voluntary participate and to terminate participation at any time, a signed consent form was obtained.

### Data collection

Given that the literature has identified that the residents’ background and admission history, and their family caregivers’ background can affect the degree of family involvement in the homes (Fukahori et al., 2006; Gaugler et al., 2003; Natan, 2008; Port, 2004), an understanding of those factors were accounted for in the demographic data collection form. For residents, data collected were age, gender, marital status, educational level, family status, care dependency (rated by the Barthel Index) (Collin, Wade, Davies, & Horne, 1988), cognitive status, length of stay and medical diagnosis were collected. For primary family caregivers, age, health status, occupation and mode of transportation used were collected. The interviews conducted had enabled residents to draw closer to their own unique experiences, and be able to express what and how they understood those experiences (Dahlberg, Dahlberg, & Nyström, 2008). Each audio-recorded interview was guided by an interview schedule and lasted for 45–60 minutes. The concepts explored in the interviews that compose the main topic of the study “Family involvement” were:

- Components of family involvement’
- Factors influencing family involvement’
- Impact of family involvement on elders’ lives, and
- Promoting family involvement strategies

After each interview, field notes of supplementary information (Burns, 1997; Morse & Field, 1995; Palys, 2008) including background data, day and time and place of interview, non-verbal behaviours and impressions of the interview were recorded. Additional documents kept by the homes that could be used to analyze the involvement of the family such as visitors’ records and residents’ going outdoors records were collected.

### Data analysis

Data collection and data analysis were undertaken simultaneously to ensure that each interview would be understood before going back onto the field to collect more data. Therefore, the audio-recorded interviews, field notes and additional documents were content-analyzed. Krippendorff (2004) defined content analysis as a research technique for making replicable and valid inference from the text. Figure 1 depicts the stepwise approach to content analysis as guided by Bengtsson (2016).

This involves repeated listening to the audio-recorded interviews and transcribed verbatim into Chinese following each interview so as to comprehend the data and to focus-in on what the participants were saying (Morse & Field, 1995). Therefore, the Chinese transcripts were prepared to download the audio-recorded interviews to facilitate easier translation into English. Backward translation was performed to ensure accuracy of the translated materials. Previous qualitative studies undertaken by the second author has always ensured accuracy in the process and procedures used to conduct translation-back translation of data so as to ensure the equivalence of meaning, and the second language is as close as possible in meaning to the original language. The guidelines used was suggested by Twinn (1997) and worked with bilingual translators who were fully competent in both languages, had a healthcare background and possessed experiences to understand the contexts in which the data were collected. Indeed, Twinn (1997) found no significant difference in the categories and themes that were generated from the Chinese and English datasets (i.e., transcripts). However, she noted that attention should be given to minor differences in the generation of themes, especially in instances when no equivalent words existed and the influence of grammatical style in the analysis. In this study, these issues were closely adhered. In conducting the analysis, the textual data were read and notes were made in the margins to identify the essences in the descriptions. Significant statements about how participants were experiencing and perceiving family involvement were identified and coded. Each new code was compared with the previous developed ones, which later became subcategories. Each code, subcategory and category was carefully examined to avoid repetition. Finally, the categories were refined to present an understanding of the phenomenon under study (Burns, 1997; Creswell, 2007; Streubert Speziale & Carpenter, 2011).

### Rigor

The trustworthiness of the study was assured by using the quality criteria as purported by qualitative researchers (Morse & Field, 1995; Polit & Beck, 2008; Streubert Speziale & Carpenter, 2011). To assure credibility, interviews were consistently conducted through the use of an interview schedule, and participants were invited to confirm the findings to be true immediately after the preliminary data analysis was completed for each interview. Dependability was assured by providing a detailed report of each stage of the research process. Clear documents relating to field notes, audio recordings and records of data analysis were carefully managed for easy data management and retrieval. Transferability of findings to other settings was achieved by providing concise background information of the participants and the research context.

## Results

### Components of family involvement

This category described residents’ experiences of three areas in which family members could be involved in their lives at the homes. These were social companionship, physiological care and support, and advocator of better care. *Social companionship* was described as finding ways to stay socially in touch with family members, either chatting to them face-to face in the home or communicating on the phone if they were unable to visit. Sometimes family members would share family affairs with the residents and would remind them to maintain living healthily. They would spare personal time to accompany the residents to watch TV, have meals together and do some physical or occupational therapies (physical exercise, Chinese handwriting and handicraft). Residents spoke about family members accompanying them to participate in competitive activities, festive celebration parties, and personal meaningful parties. One resident recalls an unforgettable family experience:

*“It was my golden wedding anniversary. The home held a party for me and my wife. My daughter, son-in-law and grandchildren came and celebrated with us. We cut a cake and ordered some food to eat in the pantry. It was very memorable.”* (C*04)*

While family members frequently enquired about residents’ moods, health status and daily living condition, this resulted in the residents not wanting to share a part of themselves with them:

*“If I tell them to come, they’ll come. Now they know I’m fine, they say to use the phone so I call my daughter every night. She says, ‘Do you have any problems?’ I tell her about my leg pain. She tells me not to go out and take more bedrest. How can I always rest on the bed? I just tell her little now.”* (C08)

Other social companionship activities that occurred outside of the home included the regular weekly or monthly visits to the tea restaurant with the extended family members. Such gatherings were a delight for residents who were grounded at the home. Activities also included taking them for outdoor walks or a ride in the car. On some occasions, arrangements were made for the residents to take home leave or go on a family trip. For example, residents would be invited as the head-of-the household to participate in family weddings and names’ giving ceremony for their grandchildren.

*Physiological care and support* was another way in which family members were involved in the residents’ lives when they gave them something they liked or wanted, although residents claimed that living necessities had already been provided by the homes. Most residents described family members bringing in foods such as soup, fruits, snacks, preserved dishes and nutritional supplements. Other bought-in items were soft clothing, quilt, leisure books, pain relief patches and simple to prepare herbal sachets. Most elders expressed that family members were not expected to do any hands-on bodily care because staff would help them. However, some close family members helped to apply ointment, attach pain relief patches, massage stiff fingers, change bodily position, make bed and fold clothes for them.

Last, *advocator for better care* was regarded by residents as another way of involving the family. For instance, family committee was recognized by residents as a forum for representing them in the promotion of better resident care. It was aimed at providing a formal and direct communicative platform for residents, family members and the home:

*“My wife could directly tell the home’s director what could be improved because she’s on the family committee. This committee collects comments and suggestions from family members to improve the services.”* (C04)

To advocate for health and well-being of residents, family members would accompany them for medical consultations as they were too old to understand their health condition:

*“They worry (since I got cancer) I may not fully understand what the doctor says. Although staff can go with me, daughter brings me to directly speak to the doctor.”* (C02)

### Factors influencing family involvement

The second category presents a number of factors that encouraged or discouraged family involvement at the homes. A factor mentioned by most residents was the livelihood of family members. They were either busy at work, studying or doing housework in the weekdays, and were only available to visit during the weekends or on holidays. Only the retired family members had more time to visit. The financial status of family members also influenced the degree of family involvement. Indeed, involving the family was perceived to be asking them to spend money on them. By being less involved in the older person’s lives, money could be saved:

*“She (wife) has no time to spare and spends less time coming here because she works. I’ve told her to call me when she can’t come. She said if she called, both of us would spend $1 on the mobile phone fee. Hey, what can I do?”* (C01)

Health status and health condition of residents was another factor for consideration, and additional time was often given to visit them when they were ill or had deteriorating health. However, when health conditions improved, the physical presence of family members would lessen and replaced by phone calls. Conversely, residents would discourage family members to come when they knew they were ill or suffered from physical discomfort or diseases which made it difficult to go or spend time at the homes:

*“My wife suffers from rheumatoid arthritis on her legs, and suffers a lot when she comes. It’s difficult to walk so I tell her not to come very often.”* (C05)

The *accessibility of family visits* was described as an influential factor affecting family involvement. For family members who conveniently lived, worked and studied near the homes, visitation would not be an issue. Correspondingly, long travelling and inconvenient transportation could reduce family involvement as described below:

*“I don’t want them to spend money and time to come here. For example, my daughter spends about HK$500 on ferry rides for each visit so she comes monthly. Another daughter works nearby and she comes here on foot after work.”* (C03)

Residents felt that more collaborative effort was required from family members to renegotiate the operations and routines of homes owing to the busy lives they lead. For these two homes, visiting hours seemed reasonably long—from 08:00–20:00 in one home to 13:00–19:00 on weekdays and from 11:00 to 19:00 on weekend in the other home. Dissatisfaction arising from interrupted family visits owing to the highly preoccupied lives of family members and their failure to abide to the regulation for visiting was raised, as one said:

*“The visiting hours are quite long but my son has to study and leaves the university at 7:00pm, and arrives at 8:00pm. The visiting time has ended. If there’re extended, it‘ll affect others sleeping time, eh?”* (C05)

The last factor was the strength of the *resident–family relationship* in determining the degree of family involvement. Some residents mentioned that when family members were involved at the home, this demonstrated caring, loving and supportive family relationship. Such occasions and meaningful interactions were times to enjoy and treasure. Participants also speculated and rumoured that poor family involvement might be attributed to poor family relationship:

*“Why is there someone living here for 2-3 years but no family members have visited? It was because of a poor family relationship! He punished and beat his son in the past! So his wife and children don’t come to see him now!”* (C04)

### Impact of family involvement on elders’ lives

An impact arising from family involvement was described as *being there and supportive*. This was a dominant phrase that was heard from the residents when family members were around. Apart from the rich-positive emotions of feeling happy, delighted, excited, impressed and comforted that were described, physical presence of family members could relieve psychological stress and promoted well-being that non-family members could not do:

*“We’ve (wife and I) stood together to face many difficulties. These (family) visits have given me support. Support from other people is not as great as from the family because we’ve been together for many decades, eh? It’s difficult to say, she’s healthier than I; life is unexpected. So I think I’ve earned a day when we can see each other.”* (C04)

A sense of worry, loss and disappointment was described when family members would discontinue or withdrew their involvements which were out of the residents’ expectations:

*“If my wife didn‘t come, I couldn‘t sleep for a whole night. She comes every 2-3 days. Last time she returned to her hometown for a few days without telling me. I worried so much…waited for her…then I was relieved when she came.”* (C01)

The extra physiological support for food, clothing and other living necessities from family members who were present was a symbol of “family cares” they live better lives at the homes:

*“My daughter bought me some snacks as dinner was served at 5pm and I ate little. I was hungry by midnight. I told her about that! She cares for my living, how I was eating and whether I was comfortable! She also bought a duvet to keep me warm!”* (C10)

Family involvement resulted in improved communication and allowed them to keep abreast with family affairs in order to maintain the stable family relationship. They understood and appreciated that family members had done lot for them (even overcommitted their responsibilities) to continue caring for them at the homes. It was a way for adult children to maintain kinship and showed filial piety:

*“He’s very filial. Others would leave when they realize that supporting older parents can cost so much money. He’s not my son, only a fostered child. He’s willing to look after me although he earns only a few thousand dollars.”* (C06)

For highly dependent residents, they perceived family involvement as being burdensome on the family. Nevertheless, it was important to seize the day—to treasure the moments of family togetherness when it did occur, especially all encounters enabled them to develop and improve relationships among family members in the long-term. Certainly, this was the case for older couples (who now live separately) to still uphold their marital relationship by accompanying and supporting each other whenever possible:

*“I shared a cake with all the residents here. I wanted to let them know my wife and I stood together. Her visits are the most precious moments for me. I don‘t know who’ll pass away first. It’s good we can meet one more day.”* (C04)

In fact, the mere supportive presence of family members had enabled them to *stay connected with the outside world*. This was something residents looked forward to when family members acted as a bridge to help them to further enrich their mundane residential lives. Life was more colourful and dignified when the family was around, especially when they participated in family-led outdoors activities, as illustrated:

*“Sometimes we go to the Golden Dragon Restaurant for dim sum. My eldest son and grandson drive me there as they know I like dim sum.”* (C09)

*“Life in the home is boring. My daughter comes and arranges activity for me. It’s brought me to see a different world*.” (C08)

Other residents also supported family involvement in the home as a good approach to promote better lives, and therefore moving towards a home-like ambience, as mentioned:

*“It seems I‘m staying in my home. The staff and nurses are very friendly. I mean the home has provided home-like environment and services. If there‘re family members involved, it‘ll be more home-like. That would make me live more comfortably.”* (C02)

### Promoting family involvement strategies

The data revealed that residents were generally satisfied with the current way their family members were involved at the homes. When enquired about the expectations for further engaging the family, they could only provide two concrete suggestions. For the over-busy family members who were most likely to be physically absent and not involved with the homes, high understanding to give them time to live their lives was expressed. Yet, they would expect regular phone call exchanges from family members as this was regarded as an efficient and effective way to stay in touch:

*“He (nephew) does not visit often. But he’d phone me. That‘s fine! He needn‘t come. If I phone him to come to see me, it seems that I don’t understand him—they should have their own lives!”* (C07)

Organizing home-led activities and gatherings were also good suggestions to try and involve the family members if they had time. Such events could promote appreciation, interactions with family and staff, and strengthen family relationships, as described below:

*“I hope my wife would come and participate in more activities. She’ll know more about resident‘s life and current practices. When she knows something good has been done, she’s relieved.”* (C04)

## Discussion

The findings of this study revealed that family involvement can contribute to providing social companionship, physiological care and support, and advocator of better care. Here, these are referred to as individual tailor-made necessities of daily living; fulfilment of a sense of love and belonging; maintaining and protecting family relationship and social connection in order to continue to lead satisfying lives at the homes. The Chinese society refers to four essential requirements of human life: clothing, food, housing and transportation as necessary for the human-being. This study found that residents reliance on family providing support for living necessities support, especially for food, an important part of settling into residential home living. A great concern for the family was to continue the responsibility to oversee the residents’ health and well-being. This finding was similar to previous findings (Commonwealth of Australia, 2013; Tsai & Tsai, 2012), and evidently there was a reliance on the younger family members to communicate with health care providers owing to their old age and lower educational level.

Several factors affecting the degree of family involvement in the homes were identified in this study. Those family members who were not so busy with their lives, lived closer to the homes and had a good relationship with the resident tended to be more involved in the homes. However, less family involvement was found in those who were over-busy with their lives, had financial difficulty, lived far away from the home, inconvenient transportation, had poorer resident–family relationship, and in poor health. Despite these less favourable factors that can lessen family involvement as already mentioned in previous studies (Fukahori et al., 2006; Natan, 2008; Port, 2004), this study highlighted that the residents state of health and illness would take precedence over these unfavourable factors. Indeed, those who were ill or had worsening health would receive more family concerns and contacts. Therefore, it would seem that greater family involvement could be due to the family being hopeful of the resident’s recovery, ensuring the continuity of ongoing care, and possibly making up for the guilt of spending less time to take care of them (Bauer, 2006; Bern-Klug & Forbes-Thompson, 2008; Tsai & Tsai, 2012). Family members provide support for their elders and occupy ambiguous positions in long-term residential care. Previous study identified good practices in promoting meaningful family involvement upon admission to residential home until the final stages of the residents’ lives. Greater appreciation of relational care work, teamwork, appropriate physical spaces and time for effective communication are facilitators for reducing conflicts and maintaining family–staff relationships to support the well-being of residents (Barken & Lowndes, 2018).

Furthermore, resident–family relationship was a core value influencing the continuity of family involvement. Such finding is congruent with prior studies that found poorer relationship between resident–family–staff could affect negatively on the frequency and duration of family visit (Fukahori et al., 2006; Gaugler et al., 2003; Natan, 2008; Port, 2004). In this study, the long-visiting hours at the homes were unhelpful for some family members whose preoccupation with work and study commitments were working against the regulated visiting hours of homes, and thereby impeded visits to see the residents. In Macao, transportation difficulty and distance to travel to the homes are not major problems as this is a small city that is equipped with good and convenient traffic networks. A suggested approach to consider would be to develop a family caregivers–staff partnership forum whereby an exchange of caring knowledge can be passed onto the family carers, and likewise exchange this to broaden understanding of the difficulties family members are facing with fitting in with the home’s policies and practices.

For the Chinese family, family members are expected to commit to filial piety which includes respecting older family members, providing financial support, treating them with dignity, and enhancing their self-worth (Chao & Roth, 2005; Creswell, 2007). Residents in this study considered family involvement as continuity of caregiving and relationship maintenance. This finding is congruent with other studies in both Chinese and overseas society (Chao & Roth, 2005; Creswell, 2007; Kellet, 2007; Whitaker, 2009). Residents in this study claimed that they did not want to bother or even burden their family members as they understood their busy lives. This finding not only support previous studies, but also reflects elder’s love for their close relatives (Cahill, Lewis, Barg, & Bogner, 2009; Reid & Chappell, 2017). In this process of involving the family, a sense of love, belonging, respect and dignity are achieved. These positive psychosocial and emotional achievements are similar to those of previous studies in western countries (Bern-Klug & Forbes-Thompson, 2008; Buckley & McCarthy, 2009; Whitaker, 2009). Indeed, the ability to connect with the community and communicating with the outside world were ascertained in this study which reemphases further inputs from the family.

This study found that residents usually encountered challenges in meeting their daily care needs, such as managing the medication and using the telephone. This is consistent with a previous study that revealed the different types and amount of family support including physical care, financial support, and monitoring the quality of staff care, but many residents always expected more (Song, Scales, Anderson, Wu, & Corazzini, 2018). A study conducted in Japan (Yamazaki & Kawahara, 2018) investigated the structure of family practices in multifunctional long-term care in a residential home. Here, family members played a part in the residents’ life by flexibly responding to sudden circumstances. The staff at the homes were able to build relationships with the family members by updating them during case intake and in regular meetings, such as providing past medical information, focusing on ways to improve the residents’ future, and connecting them to other professionals. Therefore, family members experienced more support after older people were placed in the homes.

There is a significant effect of the presence and characteristics of family caregivers on the use of long-term care for older people. Studies have indicated that residential care home was used more frequently when older people had no family caregiver, had a spouse using home healthcare services, or they had intensive care needs requiring hired care workers (Chou, Kröger, & Pu, 2015). Another study revealed that over one-half of primary caregivers decided to place their family elders in a nursing home due to their incapability to provide care, rather than the decline of physical or cognitive functions of elders (Kim, Cho, & Lee, 2013; Tamiya, Chen, & Sugisawa, 2009). Indeed, the increasing unavailability of family members, various benefits of residential care and financial situations are major considerations for the changing attitude to place older people into institutions (Zhan, Feng, Chen, & Feng, 2011). This suggests that the traditional family-based caregiving model may no longer meet the needs of Chinese families; and this has witnessed an increasing demand for residential care for older people (Reid & Chappell, 2017).

### Limitations of study

Some limitations of this study are highlighted. One limitation is that all participants were selected from government-sponsored homes. In Macao, homes subsidized by the government generally have better resources to support activities and encourage family involvement than in private homes. The inclusion of other private homes would offer a more comprehensive picture to understand the influences of social background and financial status on family involvement in long-care care institutions. Additionally, residents were nominated by the home staff for the researcher’s consideration, and thus may have some influence on how the family–staff relationship was perceived by the residents. In relation to resident–family relationships, this study painted a more positive view of the loving and caring family. The study did not provide data that indicated dysfunctional and abusive families, and therefore there is a scope to further explore the value of family involvement in these circumstances.

### Implications of study

The findings can be used to improve practices, and inform front-line health care providers’ and family members’ understanding of Chinese older residents’ perceptions about family involvement in residential care homes. The identified factors influencing family involvement can be used as a reminder to all caregivers who take care of older people to maintain and utilize those promoting factors and to avoid or overcome those barriers in their daily care. Components of family involvement may be appreciated in other geriatric care settings, and provides indicators for improving family involvement in residential care home. For the impacts of family involvement on the residents’ lives in the homes, findings support the need to develop interventions to maximize their inputs in the homes, and thereby recognizing the family as a caring partner in geriatric long term care settings. Findings also advocate allocating resources and adjusting multidisciplinary care plan to implement more interventions to promote family involvement in homes in the Chinese community, and in Macao. Findings presented insights for geriatric care providers to give thoughts to designing family-centred activities that are workable and suited for the family carers. It is a high time to recognize the positive contributions and potential partners family carers can make to actively promote the wellbeing of our seniors in residential care settings. Ideally, it is still the family that can help to facilitate the greatest degree of blessing, achievements and longevity of older residents.

## Conclusion

This study revealed four categories to understand Chinese older residents’ perceptions regarding family involvement in residential care homes of Macao. Four essential requirements of human life in Chinese culture: clothing, food, housing and transportation, are necessary for human being. This study found residents regarded the family as providing support for living and necessities, especially for food, as an important part of living well in residential care. These Chinese older people expected their children to care for them in their old age and they believed the younger family members to be their representative to protect and care for their health in the homes. Residents relied on their younger family member to communicate with the healthcare providers because of their old age and low educational level. Older residents claimed that they do not want to bother or even burden their family members to be involved in their lives in the home as they understood family members’ busy lives outside of the home. Indeed, while filial piety is important in the Chinese culture, older residents consider family involvement to be a continuity of family caregiving and improving that family relationship even though the family care arrangement is now different.
